# Twelve-month outcomes of single-step transepithelial photorefractive keratectomy for moderate hyperopia and hyperopic astigmatism

**DOI:** 10.1186/s40662-023-00327-4

**Published:** 2023-03-01

**Authors:** Mahmoud Abdel-Radi, Mahmoud Rateb, Mohamed G. A. Saleh, Mohamed Omar M. Aly

**Affiliations:** Department of Ophthalmology, Assiut University, Assiut University Hospital, 6th Floor, 71516 Assiut, Egypt

**Keywords:** Single-step PRK, StreamLight PRK, Hyperopia, Hyperopic astigmatism, Hyperopic PRK

## Abstract

**Background:**

Conventional mechanical or alcohol-assisted photorefractive keratectomy (PRK) techniques for correction of hyperopia and hyperopic astigmatism were associated with inconsistent results. The aim of this study is to evaluate the 12-month visual and refractive outcomes of the relatively new single-step transepithelial photorefractive keratectomy (TE-PRK) for moderate hyperopia and hyperopic astigmatism.

**Methods:**

This is a prospective interventional study. Forty-eight eyes of 30 patients with moderate hyperopia or hyperopic astigmatism with a cycloplegic spherical equivalent refraction (SEQ) between 2.0 and 4.5 diopters (D) underwent single-step StreamLight® TE-PRK using EX500 excimer laser (Alcon Laboratories, USA). The main outcome measures were recorded at 6 and 12 months postoperatively including assessment of logarithm of the minimum angle resolution (logMAR) uncorrected and corrected distance visual acuity (UDVA, CDVA), cycloplegic refraction, corneal topographic changes as well as post-PRK peripheral haze grading.

**Results:**

The mean preoperative cycloplegic SEQ was significantly reduced from 3.21 ± 0.61 D to 0.35 ± 0.04 D and 0.41 ± 0.04 D at 6 and 12 months, respectively (*P* < 0.001). The mean preoperative UDVA significantly improved from 0.53 ± 0.02 logMAR to 0.07 ± 0.01 logMAR and 0.08 ± 0.01 logMAR at 6 and 12 months, respectively (*P* < 0.001) while the mean preoperative logMAR CDVA showed non-significant change over time throughout the study (*P* = 0.135). At the end of the study, 41 eyes (85.4%) achieved UDVA of 20/25 or better and no eye lost any lines of CDVA. Thirty-eight eyes (79.1%) had a postoperative cycloplegic cylinder of 0.5 D or less at 12 months. The mean preoperative mean keratometry showed significant increase at 6 and 12 months postoperatively (*P* < 0.001) while there was no significant change between the two postoperative visits denoting topographic stability (*P* = 0.058). The mean postoperative Q value at 6 and 12 months showed a significant prolate shift (*P* < 0.001). No haze was observed in 62.5% and 85.4% of the enrolled eyes at 6 and 12 months, respectively.

**Conclusions:**

Single-step StreamLight® TE-PRK for moderate hyperopia and hyperopic astigmatism achieved acceptable visual and refractive outcomes.

*Trial registration*: (Clinicaltrials.gov): NCT05261685, 2 March 2022, retrospectively registered, https://clinicaltrials.gov/ct2/show/NCT05261685

**Supplementary Information:**

The online version contains supplementary material available at 10.1186/s40662-023-00327-4.

## Background

Corneal refractive procedures for hyperopia comprised of techniques that failed to gain popularity such as intracorneal inlays, conductive keratoplasty, and automated lamellar keratoplasty due to poor predictability and possible loss of corrected distance visual acuity (CDVA) [1–3]. Femtosecond laser-assisted laser in situ keratomileusis (FS-LASIK) allowing larger flap creation suitable for peripheral ablations significantly improved the outcome of hyperopic corrections [4]. However, the encountered LASIK flap complications, ectasia and the increased possibility of epithelial ingrowth with peripheral ablations encouraged many surgeons to assess accuracy and safety of surface ablation techniques to correct hyperopia [5]. Although the initial refractive results of conventional manual or alcohol-assisted photorefractive keratectomy (PRK) for hyperopia were encouraging [6], the frequent post-PRK complications including under- or over-corrections and refractive regressions have limited the utility of conventional PRK techniques to correct hyperopia [7]. Transepithelial PRK (TE-PRK) provides an alternative technique to remove the epithelium using the excimer laser followed by stromal ablation in a two-step procedure known as phototherapeutic keratectomy-photorefractive keratectomy (PTK-PRK) [8]. Advances in TE-PRK technology have allowed refractive surgeons to remove the epithelium followed by stromal ablation in a single-step utilizing an optimized epithelial ablation profile which removes the epithelium more uniformly and precisely to prevent epithelial remnants [9]. There have been studies that evaluated the outcomes of single-step TE-PRK in correcting myopia and myopic astigmatism [10, 11]. However, the aim of the current study is to assess the 12-month visual and refractive outcomes of the single-step TE-PRK technique in correcting moderate hyperopia and hyperopic astigmatism.

## Methods

### Study design

This was a prospective interventional study conducted at Tiba Eye Center (Private practice), Assiut/Egypt. All participants were fully informed about the study and provided written informed consent. Patients with a hyperopic cycloplegic SEQ between 2.0 and 4.5 D (maximum sphere 3.0 D and/or maximum cylinder 3.0 D), a steep keratometry (Ks) ≤ 46.0 D and minimum pachymetry of 500 μm were included. Excluded patients were those who were not candidates for PRK, had Ks > 46.0 D with an expected postoperative keratometry > 48.0 D and large angle kappa as estimated by an actual Pentacam measured chord mu > 0.3 mm [12]. Those with hyperopic amblyopia whether unilateral or bilateral with CDVA less than 0.2 logarithm of the minimum angle resolution (logMAR) acuity, recent contact lens wear (less than one month), dry eye and autoimmune disorders were also excluded.

### Preoperative assessment

Complete ocular examination was carried out. Preoperative refractive assessment consisted both manifest and full cycloplegic refraction measured with auto-keratorefractometer (KR-8900: Topcon, Korea republic). If there was a difference between the patient’s manifest and cycloplegic refractions (≥ 1.0 D), surgery was postponed, and patients were instructed to change their glasses prescription until they could tolerate their full cycloplegic refraction for both distance and near activities for at least 6 months before surgery. Uncorrected and corrected distance visual acuity (UDVA, CDVA) were reported using logMAR 4 m chart (Sussex Vision, Inc., Rustington, UK). Investigative evaluation included Pentacam (Oculus GmbH, Germany) for keratorefractive assessment and anterior segment ocular coherence tomography (AS-OCT, Heidelberg, GmbH, Germany) for epithelial mapping.

### Surgical technique

All eyes were treated with the StreamLight® PRK software in WaveLight EX500 Excimer Laser (WaveLight®; Alcon Laboratories, Fort Worth, TX, USA). Postoperative emmetropia was targeted in all eyes and refractive correction was adjusted based on full cycloplegic refraction [13, 14] with no specific nomograms used. After choosing the StreamLight profile, the epithelial ablation depth was determined based on epithelial mapping in a range between 45 and 65 μm (personal communication with Alcon recommends the use of maximum epithelial thickness) while the epithelial optical zone (OZ) in hyperopic corrections is 8.0 mm as a default setting. The stromal ablation OZ was set to the standard 6.5 mm for all eyes. The total ablation zone (which is a composite ablation zone for both the epithelial and stromal circles) was automatically adjusted to 8.9 mm for both the epithelial and stromal ablations to ensure epithelial-stromal ablation matching. Initially, a drop of a preservative-free local anesthetic was instilled followed by sterilizing the periocular skin and eyelashes with 10% povidone-iodine. An eyelid speculum was inserted and gentle wetting of the cornea with Merocel sponge (Medtronic Inc., Minneapolis, MN, USA) soaked with cold balanced salt solution (BSS, Alcon Lab., Fort Worth, TX, USA) followed by gentle drying was performed. Every patient was instructed to maintain their eye fixation on a green intermittent spotlight. The eye-tracker was activated, and laser ablation was focused and centered on the center of the pupil [15]. Stream excimer laser firing was started to remove the epithelium followed by stromal ablation in a single step. The manufacturer recommends a momentary stop for 10 s on hearing 3 pop sounds marking the transition between epithelial and stromal ablations to cool down the cornea. Mitomycin C (0.02%) [16, 17] was applied mid-peripherally for 60 s followed by irrigating the stroma copiously with cold BSS. A soft bandage contact lens was applied until complete epithelial regeneration. An additional movie file shows the surgical steps in more detail (see Additional file 1). Postoperative medications included Moxifloxacin 0.5% eye drops 4 times daily for a week, Fluorometholone 0.1% eye drops twice daily for a month, preservative free artificial tears 5 times daily for 3 months and oral non-steroidal anti-inflammatory pills for post-PRK pain. One surgeon (MA) performed all the TE-PRK surgeries in the study.

### Postoperative assessment

All patients were instructed to visit the refractive center daily at the same time of the day until complete epithelial regeneration was documented by negative corneal staining using sterile fluorescein 2% strip (Medicare Inc., Mumbai, India). Visual and refractive outcomes were reported at 1, 3, 6 and 12 months. Pentacam was scheduled at 6 and 12 months. Post-PRK haze was evaluated at 6 and 12 months based on Fantes et al.’s scale [18]. 0—No haze, completely clear cornea.

0.5—Trace haze seen with careful oblique illumination.

1—Haze not interfering with the visibility of fine details of the iris.

2—Mild obstruction of iris details.

3—Moderate obstruction of the iris and lens.

4—Complete opacification of the stroma in the area of the scar, anterior chamber is totally obscured.

One ophthalmologist (MO) carried out the pre- and postoperative assessment utilizing the same tools under the same settings.

### Statistical analysis

Data were analyzed using the Statistical Package for Social Science (SPSS), version 26.0. Quantitative data were tested for normality by Shapiro-Wilk test and expressed as mean ± standard deviation (SD) or standard error (SE). One-way repeated measures ANOVA was used to identify changes over time. Post hoc test with Bonferroni correction was used for pairwise comparison between every two-time period. McNemar-Bowker test was used to compare proportion of haze grading. Spearman’s correlation was used to explore correlations. The level of significance was set at *P* value < 0.05.

Sample size was calculated using the G power software version 3.1.3 using F test for repeated measures ANOVA and comparing difference in mean UDVA/SEQ preoperatively, 6 and 12 months postoperatively. Hypothesized effect size = 0.18, alpha error probability = 0.05, power = 0.8 (1− beta error probability), number of measurements was 3. Correlation among repeated measures was 0.5; non-sphericity correction *e* was 1. Therefore, the required sample size was 52 eyes. We collected 58 eyes of 38 patients and dropout occurred in eight patients. The final reported sample size that completed the planned follow up period was 48 eyes of 30 patients.

## Results

### Preoperative data

Forty-eight eyes of 30 hyperopic patients were included. Patients had a mean preoperative manifest SEQ of 2.96 ± 0.53 D and a mean preoperative cycloplegic SEQ of 3.21 ± 0.61 D with a mean manifest-cycloplegic difference of 0.25 ± 0.04 D. Table 1 presents the baseline characteristics of patients.

**Table 1:** Patient demographics and preoperative data

Parameters	Value
No. of eyes	48
No. of patients	30
Age (years), mean ± SE (range)	38 ± 1.24 (24 to 42)
Gender (female/male)	(17/13)
LogMAR UDVA (mean ± SE)	0.53 ± 0.02
LogMAR CDVA (mean ± SE)	0.02 ± 0.01
Manifest SEQ (D), mean ± SE (range)	2.96 ± 0.53 (2.00 to 4.25)
Cycloplegic SEQ (D), mean ± SE (range)	3.21 ± 0.61 (2.00 to 4.50)
Cycloplegic sphere (D), mean ± SE (range)	2.75 ± 0.05 (1.75 to 3.00)
Cycloplegic cylinder (D), mean ± SE (range)	0.93 ± 0.11 (0.00 to 3.00)
Pachymetry (μm, mean ± SE)	540.4 ± 5.6
Km (D, mean ± SD)	42.52 ± 1.19
Q value (mean ± SE)	− 0.21 ± 0.01
Central epithelial thickness (μm), mean ± SE (range)	52.81 ± 0.52 (50 to 56)
Calculated depth of ablation (μm), mean ± SE (range)	60.83 ± 4.75 (37 to 101)
Scotopic pupil (mm, mean ± SE)	5.82 ± 1.40
TBUT (seconds, mean ± SE)	12.1 ± 0.32

*SD* = standard deviation; *SE* = standard error; *CDVA* = corrected distance visual acuity; *D* = diopters; *Km* = mean keratometry; *LogMAR* = logarithm of the minimum angle resolution; *SEQ* = spherical equivalent refraction; *TBUT* = tear film break-up time; *UDVA* = uncorrected distance visual acuity

### Visual outcome

**Fig. 1:**
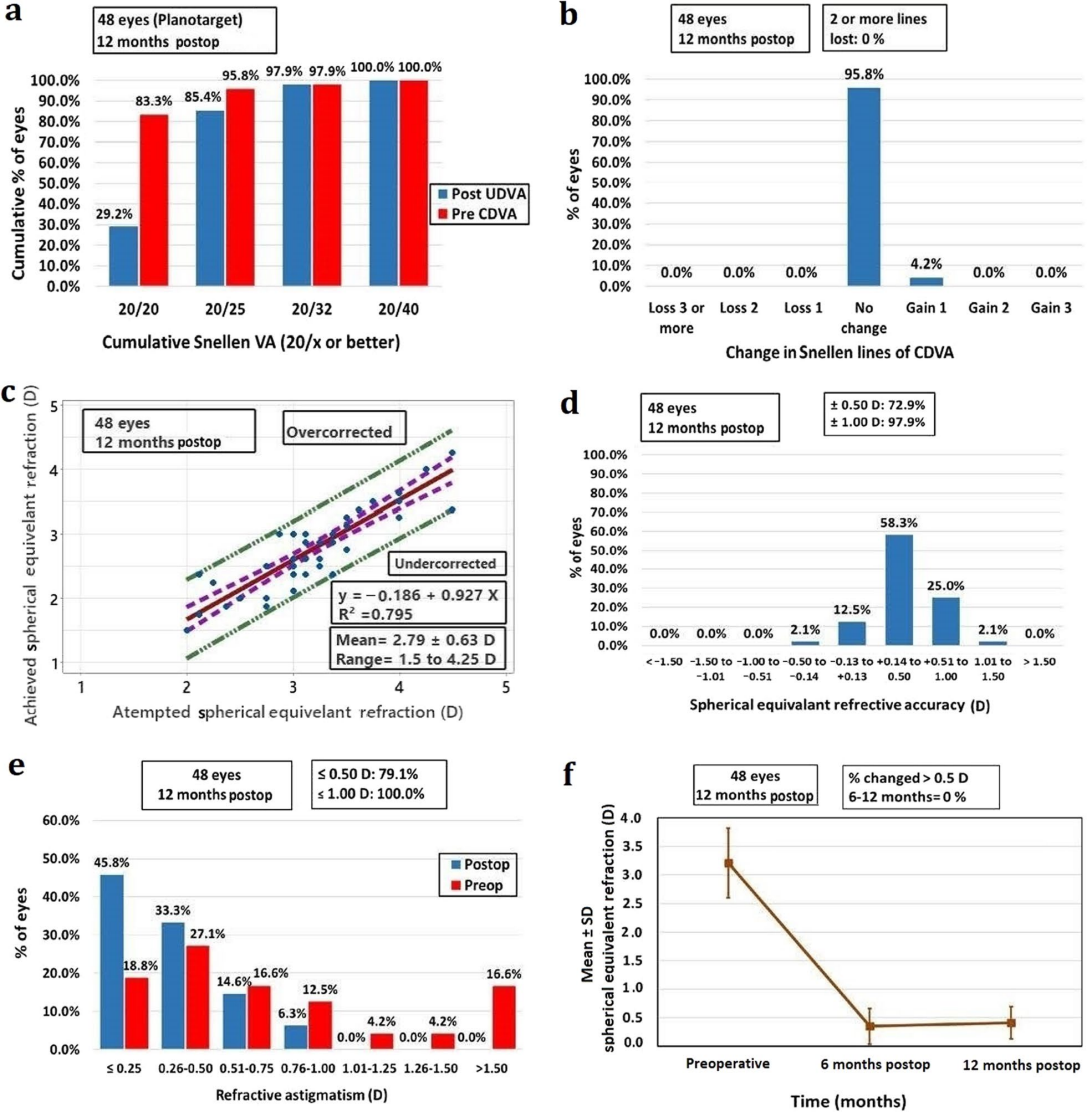
Standard graphs reporting refractive outcomes for single-step transepithelial photorefractive keratectomy (TE-PRK) for moderate hyperopia at 12 months postoperatively. **a** Uncorrected distance visual acuity (UDVA); **b** Change in corrected distance visual acuity (CDVA); **c** Attempted *vs.* achieved spherical equivalent; **d** Spherical equivalent refractive accuracy; **e** Refractive astigmatism; **f** Stability of spherical equivalent refraction; Postop, postoperative; Preop, preoperative

The mean preoperative logMAR UDVA significantly improved from 0.53 ± 0.02 to 0.07 ± 0.01 and 0.08 ± 0.01 at 6 and 12 months, respectively (*P* < 0.001, Table 2) while there was no significant change between 6 and 12 months (Table 2). At the end of the follow-up period (12 months), 14 eyes (29.2%) achieved UDVA of 20/20, 41 eyes (85.4%) achieved UDVA of 20/25 or better and all eyes (48 eyes) achieved UDVA of 20/40 or better as shown in Fig. 1a.

**Table 2:** Visual, refractive and topographic outcomes of single-step transepithelial PRK for hyperopia

Variables		Preoperative	Postoperative 6 months	Postoperative 12 months	*P* value*	*P*^a^	*P*^b^	*P*^c^
UDVA (logMAR)	Mean ± SE	0.53 ± 0.02	0.07 ± 0.01	0.08 ± 0.01	** < 0.001**	** < 0.001**	** < 0.001**	0.999
	Range	0.3 to 0.8	0.0 to 0.2	0.0 to 0.3				
CDVA (logMAR)	Mean ± SE	0.02 ± 0.01	0.010 ± 0.007	0.010 ± 0.007	0.135	NA	NA	NA
	Range	0.0 to 0.3	0.0 to 0.2	0.0 to 0.2				
Manifest SEQ (D)	Mean ± SE	2.96 ± 0.53	0.33 ± 0.04	0.38 ± 0.03	** < 0.001**	** < 0.001**	** < 0.001**	0.280
	Range	2.00 to 4.25	− 0.25 to 0.75	− 0.25 to 1.0				
Cycloplegic SEQ (D)	Mean ± SE	3.21 ± 0.61	0.35 ± 0.04	0.41 ± 0.04	** < 0.001**	** < 0.001**	** < 0.001**	0.459
	Range	2.0 to 4.5	− 0.25 to 1.0	− 0.25 to 1.12				
Cycloplegic sphere (D)	Mean ± SE	2.75 ± 0.05	0.16 ± 0.05	0.22 ± 0.04	** < 0.001**	** < 0.001**	**0.001**	0.234
	Range	1.75 to 3.00	− 0.5 to 0.75	− 0.5 to 0.75				
Cycloplegic cylinder (D)	Mean ± SE	0.93 ± 0.11	0.35 ± 0.03	0.39 ± 0.04	** < 0.001**	** < 0.001**	**0.001**	0.999
	Range	0.00 to 3.00	0.00 to 0.75	0.00 to 1.00				
Km (D)	Mean ± SD	42.52 ± 1.19	44.76 ± 1.31	44.74 ± 1.29	** < 0.001**	** < 0.001**	** < 0.001**	0.058
	Range	40.3 to 45.8	42.2 to 47.7	42.2 to 47.7				
Q value	Mean ± SE	− 0.21 ± 0.01	− 0.90 ± 0.02	− 0.89 ± 0.02	** < 0.001**	** < 0.001**	** < 0.001**	0.785
	Range	− 0.11 to − 0.34	− 0.62 to − 1.34	− 0.63 to − 1.34				
	Difference	NA	− 0.69 ± 0.01	− 0.68 ± 0.01	NA	** < 0.001**	** < 0.001**	0.940

*P* values that are less than 0.05 (*P* value < 0.05) were considered significant and provided in bold

*SD* = standard deviation; *SE =*  standard error; *UDVA =*  uncorrected distance visual acuity; *CDVA =*  corrected distance visual acuity; *LogMAR =*  logarithm of the minimum angle resolution; *SEQ =*  spherical equivalent refraction; *Km =*  mean keratometry; *D =*  diopter; *NA =*  not available

^*^One-way repeated measures ANOVA test with Bonferroni correction. ^a^*P*: preoperative vs. 6 months postoperative. ^b^*P*: preoperative vs. postoperative 12 months. ^c^*P*: postoperative 6 months vs. 12 months

The mean preoperative logMAR CDVA showed non-significant change over time throughout the study (*P* = 0.135, Table 2). None of the included eyes lost any Snellen lines of CDVA and two eyes gained one line (4.2%) at the final follow-up visit (Fig. 1b).

#### Efficacy and safety

**Fig. 2:**
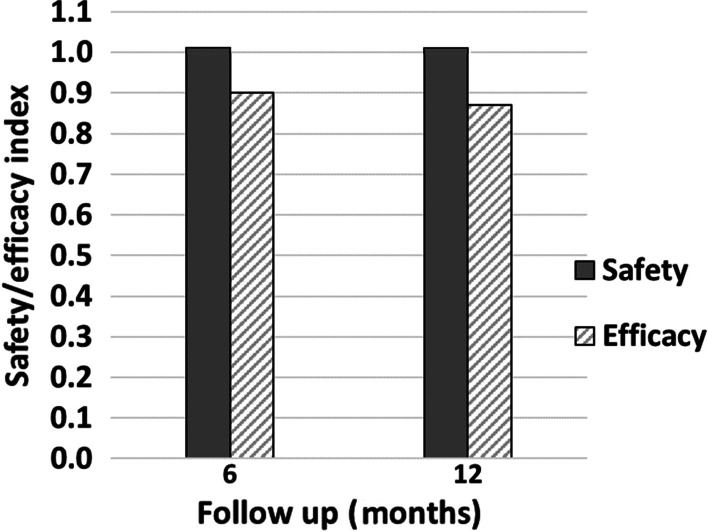
Safety/efficacy index graph. Safety and efficacy indices at 6 and 12 months following single-step transepithelial photorefractive keratectomy (TE-PRK) for moderate hyperopia

The efficacy index of the procedure (the ratio of the mean postoperative UDVA to the mean preoperative CDVA) was 0.90 and 0.87 at 6 and 12 months, respectively. The safety of the procedure was high with a safety index (the ratio of the mean postoperative CDVA to the mean preoperative CDVA) of 1.01 at both follow-up visits as illustrated in Fig. 2.

### Refractive outcome

#### Spherical equivalent refraction (SEQ)

The mean cycloplegic SEQ was reduced from 3.21 ± 0.61 D preoperatively to 0.35 ± 0.04 D and 0.41 ± 0.04 D postoperatively at 6 and 12 months, respectively (*P* < 0.001, Table 2) while there was no statistical difference between 6 and 12 months (*P* = 0.459). The predictability of the surgical procedure at 12 months is presented in Fig. 1c showing the relationship between attempted and achieved cycloplegic SEQ with a coefficient of determination r^2^ = 0.795. At 12 months, the accuracy of the postoperative cycloplegic SEQ within ± 0.5 D of emmetropia was achieved in 35 eyes (72.9%) as displayed in Fig. 1d. Stability of the postoperative cycloplegic SEQ was documented as illustrated in Fig. 1f.

#### Astigmatism

The mean preoperative cycloplegic cylinder (expressed in a plus cylinder notation) significantly improved from 0.93 ± 0.11 D to 0.35 ± 0.03 D and 0.39 ± 0.04 D at 6 and 12 months following surgery, respectively (*P* < 0.001, Table 2). Thirty-eight eyes (79.1%) had a postoperative cycloplegic cylinder of 0.5 D or less at 12 months as shown in Fig. 1e.

Figure 1 shows the six-standard graphs for reporting the 12-month refractive outcomes for the single-step TE-PRK for moderate hyperopia.

### Topographic outcome

**Fig. 3:**
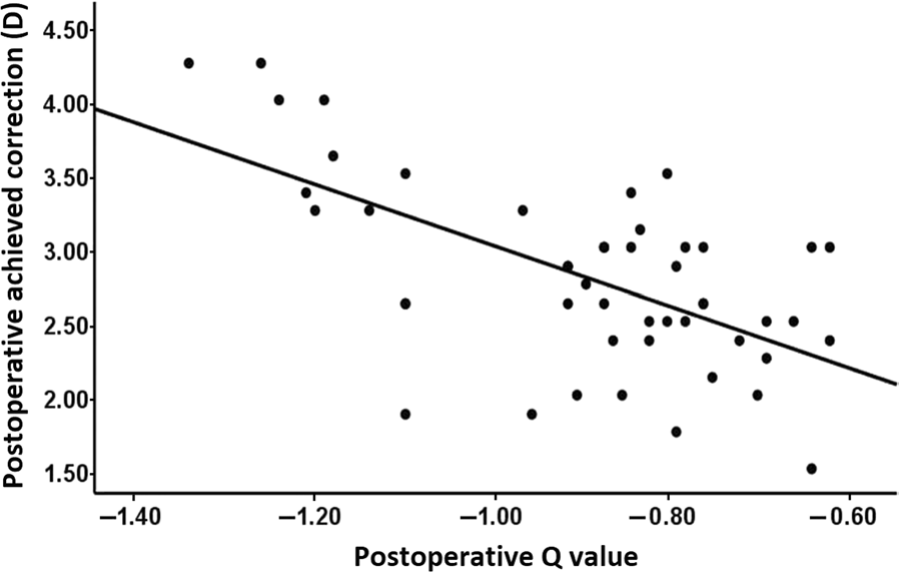
Postoperative achieved correction and Q value. Correlation between the postoperative achieved correction and Q value at 12 months postoperatively

The mean preoperative mean keratometry (Km) was 42.52 ± 1.19 D which increased significantly to 44.76 ± 1.31 D at 6 months and 44.74 ± 1.29 D at 12 months (*P* < 0.001, Table 2) with a non-significant change between 6 and 12 months (*P* = 0.058) denoting postoperative topographic stability over one-year follow-up. Q value assessment showed a prolate shift of all eyes as evidenced by significant increase of the mean negative Q value from − 0.21 ± 0.01 preoperatively to − 0.90 ± 0.02 and − 0.89 ± 0.02 at 6 and 12 months postoperatively, respectively (*P* < 0.001, Table 2). A significant negative correlation was observed between the achieved correction and postoperative Q value at 12 months (Spearman’s correlation, r =  − 0.514, *P* < 0.001) as demonstrated in Fig. 3.

### Complications

#### Epithelial healing

Forty-three eyes (89.5%) showed complete epithelial healing on the third postoperative day and all eyes (100%) on the fourth postoperative day.

#### Haze scores

**Fig. 4:**
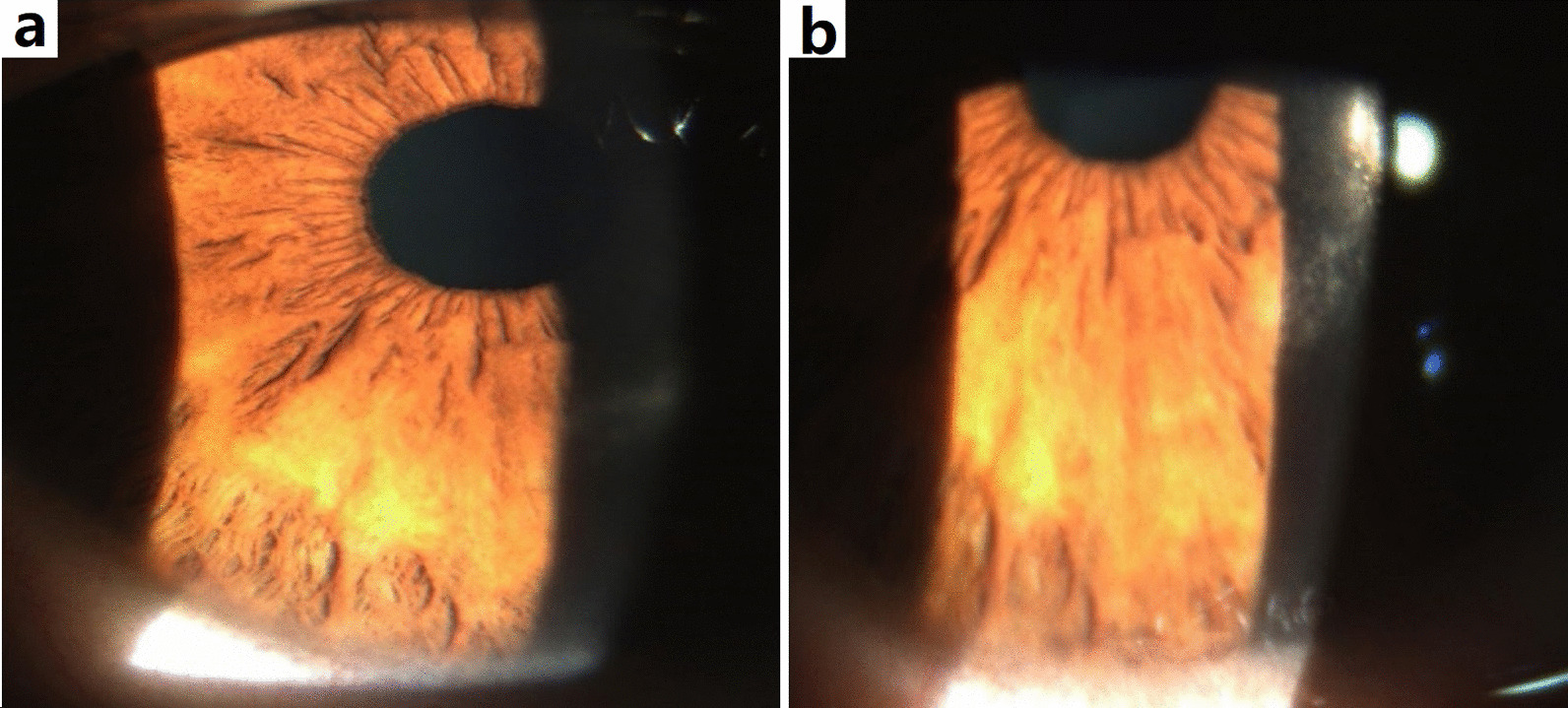
Post-PRK peripheral haze. Two slit-lamp photos of a 38-year-old case with grade 1 peripheral haze at 12 months following bilateral single-step transepithelial photorefractive keratectomy (TE-PRK) for moderate hyperopia. **a** Right eye; **b** Left eye

Zero haze was observed in 62.5% and 85.4% of the enrolled eyes at 6 and 12 months, respectively (*P* = 0.003, Table 3). None of the included patients had post-PRK haze exceeding grade 1 at 6 and 12 months (Fig. 4).

**Table 3:** Postoperative peripheral haze grading at 6 and 12 months

Haze grade	6 months postoperative No. of eyes (%)	12 months postoperative No. of eyes (%)	*P value
0	30 (62.5%)	41 (85.4%)	**0.003**
0.5 and 1	18 (37.5%)	7 (14.6%)	**0.003**

*P* values that are less than 0.05 (*P* value < 0.05) were considered significant and provided in bold

^*^Statistical significance (McNemar-Bowker test)

## Discussion

Refractive surgical options for hyperopia can be either cornea- or lens-based surgeries. The risk of intraocular surgery complications remains a main limitation of lens-based procedures [19, 20].

Corneal refractive surgery for hyperopia is challenging due to the difference between manifest and cycloplegic refractions, peripheral stromal ablation, the centration debate and frequent regression [21–23]. Advances in corneal refractive surgery have markedly improved the predictability and safety of hyperopic corrections [24].

To the best of our knowledge, one bicentric study [25] investigated the efficacy of single-step TE-PRK technique for hyperopia with Amaris 500-Hz (SCHWIND, Germany). Despite the novelty of the study, it had some limitations such as being retrospective and most importantly, the postoperative refractive assessment was based on manifest refractions that might miss possible undercorrections. Hence, this is the first study to evaluate the single-step TE-PRK technique for hyperopia with EX500 excimer laser StreamLight® technology (Alcon Laboratories, USA) in a prospective design based on cycloplegic refraction data.

In a study conducted by Adib-Moghaddam et al. [25] assessing the 12-month efficacy of single-step TE-PRK for low and moderate hyperopia, UDVA of 20/25 or better was achieved in 52.4% in the moderate hyperopia subgroup compared with 85.4% in our study. They reported the loss of one logMAR line for preoperative CDVA in 35.1% in the same subgroup at 12 months while no eye in our study lost any logMAR lines. Our relatively better distance visual outcome whether uncorrected or corrected could be attributed to the fact that the mean preoperative logMAR UDVA in our study (0.53 ± 0.02) was better than their mean preoperative logMAR UDVA in the moderate hyperopia subgroup (0.7 ± 0.07). In addition, our mean preoperative cycloplegic SEQ (3.21 ± 0.61 D) was lower than their mean preoperative manifest SEQ in the same subgroup (3.65 ± 0.13 D). On the other hand, Habibollahi et al. [26] evaluated the visual outcome of mechanical PRK for hyperopia and found that 42.9% of included eyes in their study, achieved UDVA of 20/20 compared with 29.2% in our study but they included patients with low and moderate hyperopia without subgroup stratification.

Early postoperative temporary myopic overshoot after hyperopic refractive surgery had been suggested by many studies with different interpretations such as initial overcorrection followed by later regression [27, 28] or early accommodative spasm followed by relaxation [29]. Here, we did not notice any initial myopic overshoot with a mean cycloplegic SEQ of 0.33 ± 0.09 and 0.35 ± 0.09 D at 1 and 3 months, respectively. A possible explanation is that our postoperative refractive analysis was dependent on cycloplegic rather than manifest refractions as well as our strict preoperative inclusion criterion of tolerance of full cycloplegic refraction at least 6 months before surgery. However, statistical analysis was restricted to data from 6 and 12 months follow-up visits due to the fact that epithelial remodeling and stromal tissue response in PRK continue for many months following surgery [30] and to allow for complete refractive stability [31].

Refractive regression was previously reported in earlier hyperopic PRK practice utilizing smaller treatment OZ with abrupt transition resulting in exaggerated epithelial and stromal tissue regeneration especially with high hyperopic corrections [32]. Refractive stability observed in our study could be explained by the use of a large 8.0 mm epithelial OZ with an ablation zone of 8.9 mm ensuring a smooth transition beyond the standard 6.5 mm stromal ablation circle. A study by O'Brart et al. [33] investigated the long-term refractive stability of hyperopic PRK and concluded that stability achieved at one year was maintained up to 7.5 years with no evidence of hyperopic shift. On the contrary, Wagh et al. [34] reported a significant increase in hyperopic SE between one year and 7.5 years after hyperopic PRK. However, the mean age in Wagh's cohort was 53 years, and thus the hyperopic drift was expected to result from physiologic lenticular changes rather than a true PRK regression as supported by stable keratometry.

Some studies [35, 36] do not consider pupil centration for hyperopic corrections as it would decenter the actual ablation in relation to the line of sight with resultant astigmatism induction. However, the exclusion of patients with large angle Kappa in our study and the minimally encountered post-PRK haze are important factors explaining the reasonable astigmatic outcome of our pupil-centered hyperopic PRK surgery.

The peculiar StreamLight technology could elucidate the acceptable visual and refractive outcomes of the single-step TE-PRK surgery for moderate hyperopia in the current study. The software allows utilizing a large epithelial OZ for hyperopic corrections with a large ablation zone to achieve a perfect match between the epithelial and stromal ablation profiles without abrupt transition. The single-step procedure shortens the required surgical time with a single centration applicable throughout the whole procedure. It also permits the entry of the actual epithelial thickness based on AS-OCT epithelial mapping to ensure complete epithelial removal and a refraction neutral de-epithelialization at the area of concern (personal communication with Alcon). The manufacturer recommends the entry of the maximum epithelial thickness in epithelial mapping assuming that there are no significant differences of the epithelial thickness between the center and the periphery especially at the treated OZ as supported by previous studies [37, 38].

Observing the keratometry, which is an objective method to calculate the change of refraction at the cornea [40], our results showed that the keratometric power change (preoperative/postoperative) was nearly 0.5 to 0.75 D less than the intended refractive correction. A possible explanation for this observation is that hyperopic treatments involve more transition points along the ablation diameter, produce less predictable biomechanical changes that may contribute to greater variability in corneal curvature and power than myopic treatments as concluded by Qazi et al. [43].

Topographic stability over one-year follow-up was documented in our study similar to Moawad et al. [41] who reported fewer topographic changes after mitomycin C assisted hyperopic LASIK. Gauthier-Fournet et al. [42] evaluated the effect of postoperative keratometry on the safety of LASIK for high hyperopia up to 9.5 D and found that corneas with steeper postoperative keratometries were associated with higher loss of CDVA. Significant prolate shift of the included eyes was observed in our study that is expected to produce a large amount of negative spherical aberrations following corneal hyperopic refractive procedures as reported previously [43]. However, the induction of these higher-order aberrations following hyperopic PRK has been associated with improvement in unaided near vision especially of presbyopic patients due to increased depth of focus [44].

No postoperative complications were reported in the study apart from minimal peripheral haze. A similar study [45] of alcohol-assisted PRK utilizing a larger OZ of 7.5 mm with a 10.0 mm ablation zone for correction of higher degrees of hyperopia showed similar minimal haze that could be attributed to the peripheral application of mitomycin C, cold BSS and the use of modern excimer laser ablation profiles.

Limitations of our study include the small sample size with inclusion of both eyes of some patients, the restricted inclusion of patients with small angle Kappa and the lack of analysis of postoperative aberrations. One of the main limitations of the StreamLight® PRK software in hyperopia is that it allows for a maximum hyperopic correction of 3.0 D sphere and/or 3.0 D cylinder with maximum SEQ of 4.5 D as treatment limits.

## Conclusions

Single-step StreamLight® TE-PRK is an effective, predictable and safe refractive procedure for correcting moderate hyperopia and hyperopic astigmatism. However, further studies with longer follow-up periods are required in addition to the need for software evolution to allow for directly exporting epithelial mapping data and to achieve efficient and safe correction of higher degrees of hyperopia beyond the current treatment limits.

## Supplementary Information

**Additional file 1**: **Video S1.** Single-step StreamLight® PRK for hyperopia video**.** Surgical steps of single-step transepithelial photorefractive keratectomy for hyperopia.

## Data Availability

Data are available from the corresponding author on reasonable request.

## Abbreviations

PRK: Photorefractive keratectomy
TE-PRK: Transepithelial photorefractive keratectomy
PTK-PRK: Phototherapeutic keratectomy-photorefractive keratectomy
FS-LASIK: Femtosecond laser-assisted laser in situ keratomileusis
UDVA: Uncorrected distance visual acuity
CDVA: Corrected distance visual acuity
logMAR: Logarithm of the minimum angle of resolution
SEQ: Spherical equivalent refraction
AS-OCT: Anterior segment ocular coherence tomography
OZ: Optical zone
Km: Mean keratometry
TBUT: Tear film break-up time
